# First clinical experience with a novel, mobile cone-beam CT system for treatment quality assurance in brachytherapy

**DOI:** 10.1007/s00066-022-01912-7

**Published:** 2022-03-12

**Authors:** Andre Karius, Vratislav Strnad, Michael Lotter, Stephan Kreppner, Christoph Bert

**Affiliations:** 1 Department of Radiation Oncology, Universitätsklinikum Erlangen, Friedrich-Alexander Universität Erlangen-Nürnberg, Universitätsstr. 27, 91054 Erlangen, Germany; 2 Comprehensive Cancer Center Erlangen-EMN (CCC ER-EMN), Erlangen, Germany

**Keywords:** Image-guidance, Adaptive brachytherapy, Gynecologic brachytherapy, On-site computed tomography imaging, Mobile imaging

## Abstract

**Background and purpose:**

On-site cone-beam computed tomography (CBCT) has gained in importance in adaptive brachytherapy during recent years. Besides treatment planning, there is increased need particularly for image-guidance during interventional procedures and for image-guided treatment quality assurance (QA). For this purpose, an innovative CBCT device was rolled out at our hospital as the first site worldwide. We present the first clinical images and experiences.

**Materials and methods:**

The novel CBCT system is constructed of a 121 cm diameter ring gantry, and features a 43.2 × 43.2 cm^2^ flat-panel detector, wireless remote-control via tablet-PC, and battery-powered maneuverability. Within the first months of clinical operation, we performed CBCT-based treatment QA for a total of 26 patients (8 with breast, 16 with cervix, and 2 with vaginal cancer). CBCT scans were analyzed regarding potential movements of implanted applicators in-situ during the brachytherapy course.

**Results:**

With the presented device, treatment QA was feasible for the majority of patients. The CBCT scans of breast patients showed sufficient contrast between implanted catheters and tissue. For gynecologic patients, a distinct visualization of applicators was achieved in general. However, reasonable differentiations of organic soft tissues were not feasible.

**Conclusion:**

The CBCT system allowed basic treatment QA measures for breast and gynecologic patients. For image-guidance during interventional brachytherapy procedures, the current image quality is not adequate. Substantial performance enhancements are required for intraoperative image-guidance.

## Introduction

Computed tomography (CT) is a well-established imaging modality and integral part of the imaging workflow in several medical specialties [1–9]. In recent years, particularly cone-beam CT (CBCT) has found increasing applications for interventional [1] and intraoperative [2, 3] purposes. One factor for this is that corresponding mobile devices as C‑arms [1–4] or O‑arms [5] allow working flexible at the patient, even in imaging position.

Potential for CBCT in brachytherapy is undoubted. Due to the modality’s good high-contrast capability, resolution, and geometric accuracy, CBCT allows in general distinct visualizations of inserted applicators [6]. As examples, Peters et al. [7] performed CBCT-based adaptive planning of seed implantations into the prostate. Al-Halabi et al. [8] reported respective brachytherapy planning for cervical cancer. Besides treatment planning, there is increased need also for improvements in treatment quality assurance (QA), both during the surgical procedure and throughout the therapy course. This is crucial for therapy success, since interfractional applicator position changes might have significant effects on the dose delivered to target volumes and organs at risk (OARs) [10–14]. Image-guided real-time checks of applicator positions appear essential to account for patient-specific clinical requirements and to ensure accurate applicator arrangements. CBCT represents a suitable modality for the aforementioned imaging tasks.

CBCT scanners for interventional environments usually feature mobile designs [15–18]. Concurrently, they show significant image quality limitations compared to conventional CT, especially regarding image artifacts [15–18]. The clinical use of respective devices may require more effort than three-dimensional (3D) C‑arms [19], due to larger space requirements. In this respect, a compact device design is considered important for the smooth integration in interventional workflows. Furthermore, to enable patient examinations in the lithotomy position required for intraoperative brachytherapy, a large gantry clearance is crucial. One way to combine these requirements is the usage of vast flat-panel detectors (FPDs). These enable examinations of large anatomical regions of interests (ROIs) with one single gantry rotation and non-moving patient table. In addition to CBCT, radiography and fluoroscopy can be performed with FPDs in general. The combination of these imaging modalities particularly provides the possibility of 2D–3D image registration. This has shown to enable rapid assessments of surgical outcomes such as implant stability [20, 21].

On-site CBCT enables the cross-sectional visualization of the patient anatomy with applicators in situ directly at the brachytherapy ward. This allows both treatment planning as well as intraoperative and interfractional treatment QA measures. For this purpose, we rolled out the innovative CBCT system ImagingRing m (medPhoton, Salzburg, Austria) as the first site worldwide. The clinical introduction started in February 2021 and focused on patients with breast cancer and gynecologic malignancies. We present the first clinical images and experiences.

## Materials and methods

### ImagingRing m

**Fig. 1:**
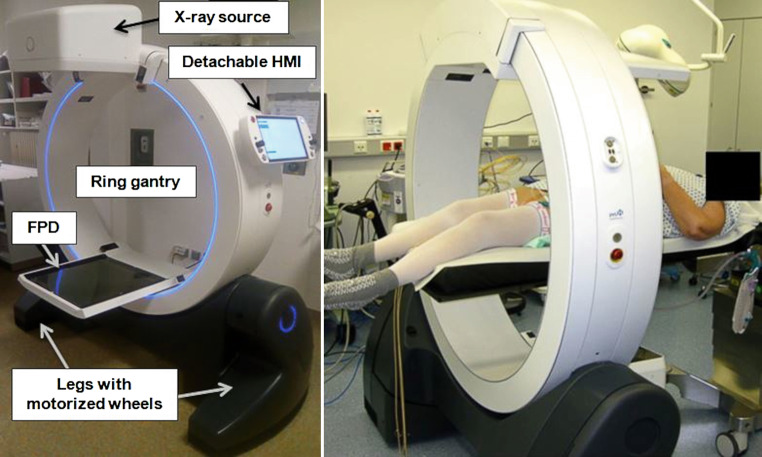
ImagingRing m (IRm) design and patient setup: The IRm is constructed of a ring gantry, on which X‑ray source and flat-panel detector (*FPD*) are mounted. The gantry is mounted to so-called legs with motorized wheels, enabling the device’s high mobility. The IRm is operated by means of the Human Machine Interface (*HMI*)

The ImagingRing m (IRm) features CBCT, radiography, and fluoroscopy as imaging modalities. It is constructed of a 121 cm ring gantry, on which X‑ray source and 43.2 × 43.2 cm^2^ flat-panel detector (FPD) are mounted (Fig. 1). Source and detector can rotate independently along the gantry, thus, allowing for non-isocentric imaging. The FPD is by default operated with a frame rate of 12 Hz and 300 µm pixel size. For detailed technical specifications of the system, please refer to its physical characterization published previously [22].

Within a so-called volume–definition workflow (VDW), four independently, dynamically collimating jaws allow non-isocentric imaging and user-controlled beam-shaping. In this VDW, anterior–posterior and lateral topograms are taken first. Based on these images, the 3D field of view (FOV) of a CBCT scan can then be specified prior its acquisition. Fully opened, the jaws provide for each projection a maximum isocentric planar FOV of 25.4 × 25.4 cm^2^. For beam quality adjustments, six different prefilters (air; 1.5 mm Al; 0.2 mm, 0.5 mm, and 1.5 mm Cu; 0.3 mm Cu BowTie) can be inserted into the beam path. Four built-in cameras enable patient monitoring during examinations.

The IRm is operated via WLAN remote-control by means of a portable so-called Human Machine Interface (HMI, Fig. 1) that is established on a Windows®(Microsoft, Redmond, Washington, USA)-based tablet-PC. Motorized wheels at the bottom of each leg (Fig. 1) as well as gearboxes enable lateral and longitudinal movements, free rotations, and ±30° gantry tilt. All movements can be performed battery-powered for up to 30 min. This facilitates changing the premises in which the system is used.

The IRm distinguishes itself from previous CBCT systems [1–5] particularly by the battery-powered mobility, tablet-PC operation, dynamic collimation, non-isocentric imaging, as well as large gantry and FPD. Prior to the device’s clinical roll-out, we conducted a profound technical characterization of its imaging performance using standard QA phantoms. We have reported our results recently [22] (please refer to this publication for detailed information). Due to the good high-contrast visualization achieved in these studies, the IRm was considered suitable for the detection of brachytherapy applicators.

### Workflow for breast/gynecologic brachytherapy

At our institution, interstitial brachytherapy as sole therapy modality for breast cancer is performed as high-dose-rate (HDR) Accelerated Partial Breast Irradiation (APBI) [23, 24]. Cervix and vaginal cancers are treated with intracavitary and interstitial techniques. Treatment is performed either in the HDR or pulsed-dose-rate (PDR) regime, depending on specific entity and case.

For gynecologic tumors, brachytherapy starts with the intrauterine, intravaginal, and/or interstitial insertion of appropriate applicators, which are chosen adapted to the prevailing tumor characteristic. This is performed under general anesthesia and guided by transrectal ultrasound. For breast brachytherapy, the implantation of flexible plastic catheters into the breast tissue is performed under radiography control. Following these steps, the irradiation is planned based on CT scans with applicators in situ. The full treatment usually lasts one week for PDR gynecologic and HDR breast patients, and a few days to weeks for HDR gynecologic patients (depending on the case).

About halfway through the treatment period, we acquire a control-CBCT with the IRm (see scheme in Table 1). Patients at high risk of applicator displacement due to repositioning are excluded. For breast patients, we perform this CBCT check to detect potential catheter arrangement variations in situ and to assess the necessity for treatment adaptions. These proceedings follow the methodologies described in detail by Kallis et al. [25].

**Table 1:** Time scheme for performing control-CBCT acquisitions

Tumor entity	Treatment modality	Slot for control-CBCT
Breast cancer	HDR, 9 × 3.8 Gy	After 4th fraction
Cervix cancer	HDR, 2 × 7 Gy	Prior to 2nd fraction
	PDR, 70 × 0.5 Gy	After 35th fraction ± 4 h
Vaginal cancer	HDR, 6 × 5 Gy	After 3rd fraction
	PDR, 70 × 0.5 Gy	After 35th fraction ± 4 h

*CBCT* cone-beam computed tomography, *HDR* high-dose-rate, *PDR* pulsed-dose-rate

For the QA of gynecologic brachytherapy, we analyze the CBCT scans immediately after acquisition in search for potential applicator position changes. Senior physician and medical physicist assess the relative distances between needles and applicators in all three main planes as well as multiplanar reconstructions. Both the distances between the individual applicator and needle tips as well as the in-plane distances between applicators and needles at the symphysis level are measured. The measured distances are then compared to the intended distances, which are documented on the (day 0) planning-CT. Moreover, we visually inspect the exact positions of needle tips and the centered location of the probe within the uterus. For patients who may be subject to swaps in afterloader transfer tubes, X‑ray markers are inserted into the applicators to verify the proper applicator–transfer tube assignment. All these factors ensure that the intended dose distribution can be delivered technically correct to the tissue. If discrepancies are found in any of the above, treatment adaption by re-planning or applicator adjustment is foreseen.

### Patient and imaging parameters

Within the first 3 months of the IRm’s clinical operation, we treated 8 breast, 16 cervix, and 2 vaginal cancer patients with the described workflow. All gynecologic patients were treated with PDR brachytherapy. Patient characteristics are listed in Table 2.

**Table 2:** Characteristics of the examined patients. Furthermore, the contrast–noise ratio (CNR) measured for each patient between catheter and surrounding tissue (for breast patients) and exemplary between uterus and bladder (for gynecological patients) are provided

Breast patients					Gynecologic patients					
ID	Age	BMI	No. of catheters	CNR	ID	Age	BMI	Cancer	Applicators	CNR
PB1	48	24	19	7.7	PG1	52	29	Cervix	Fletcher	2.4
PB2	70	29	18	5.9	PG2	36	23	Cervix	Fletcher	0.2
PB3	63	41	20	^a^	PG3	44	24	Cervix	Fletcher + 6 needles	4.8
PB4	83	24	22	9.2	PG4	40	21	Cervix	Fletcher + 3 needles	2.5
PB5	55	34	19	^b^	PG5	62	31	Cervix	Fletcher	0.2
PB6	61	31	13	8.4	PG6	78	20	Cervix	Fletcher + 3 needles	2.1
PB7	64	27	18	6.1	PG7	62	23	Vagina	Papillon template + 4 needles	3.2
PB8	63	25	20	7.1	PG8	86	20	Vagina	Prostate template + 7 needles	0.5
					PG9	82	23	Cervix	Syed template + 6 needles	0.7
					PG10	80	23	Cervix	9 Heymann applicators	2.3
					PG11	41	22	Cervix	Fletcher	3.9
					PG12	84	36	Cervix	Fletcher + 6 needles	0.5
					PG13	55	23	Cervix	Fletcher	1.4
					PG14	25	24	Cervix	Fletcher + 2 needles	1.2
					PG15	48	23	Cervix	Syed template + 4 needles	0.9
					PG16	39	23	Cervix	Fletcher + 2 needles	3.2
					PG17	61	20	Cervix	Syed template + 10 needles	3.5
					PG18	64	35	Cervix	Fletcher	1.6
Median	63	28	–	7.7	–	58	23	–	–	2.1
Range	48–83	24–41	–	5.9–9.2	–	25–86	20–36	–	–	0.2–4.8

*BMI* body mass index in kg/m^2^, *ID* identification

^a^No scan, due to technical problems

^b^No compliance with breathing commands, blurred catheter paths

The IRm did not feature scan protocols for breast and pelvis imaging sufficient for our clinical requirements nor an automatic exposure control. Therefore, the in-house development of protocols and the manual adaption of protocol parameters (prefiltering, current–time product, etc.) to varying patient characteristics was necessary. These proceedings were based on our experience and particularly on the results of the extensive physical characterization of the device [22].

All examinations were performed using the VDW. Patients were always lying on a carbon fiber table (Fig. 1). Breast patients were scanned head-first supine in breath-hold with an acquisition time of 16–18 seconds. Gynecologic patients were examined feet-first supine with flat-lying/non-bent knees (Fig. 1) and filled bladder. The median acquisition time was 40 seconds (range 19–70 seconds). Starting with the eleventh gynecologic patient (named PG11, Table 2), an abdominal belt was tightly applied and butylscopolaminium bromide was administered 15 minutes before each scan. This aimed to reduce image quality deteriorations which may result from respiration or bowel peristalsis.

### Assessment of structure differentiability

To provide a quantitative measure for the delimitability of individual tissues as target volumes and OARs, we calculated the contrast–noise ratio (CNR) for each scan. This is a decisive metric for the distinct differentiation of structures:

1 $$\mathrm{CNR}_{1,2}=\frac{| \mathrm{ROI}_{1}-\mathrm{ROI}_{2}| }{\sqrt{\frac{1}{2}\cdot \left({\sigma _{1}}^{2}+{\sigma _{2}}^{2}\right)}}$$

**Fig. 2:**
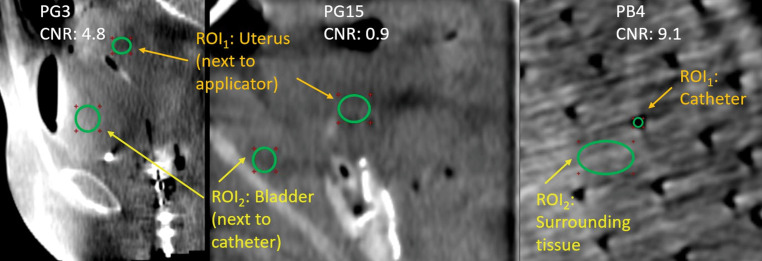
Regions of interest (*ROI*) placement for contrast–noise ratio (*CNR*) calculations, exemplarily shown for two gynecologic and one breast patient. For gynecologic patients, ROI_1_ (uterus) and ROI_2_ (bladder) were each placed adjacent to applicator and bladder catheter, respectively. Level: 60 HU, Width: 400 HU. CNR measurements were performed using RadiAnt^TM^ DICOM Viewer (Medixant, Poznan, Poland)

*ROI*_1_  and *ROI*_2_  denote the mean CT numbers, *σ*_1_ and *σ*_2_  the CT-number standard deviations of the considered ROIs, respectively. For breast patients, CNR was measured between catheters (*ROI*_1_ ) and surrounding tissue (*ROI*_2_ ) (Fig. 2). For gynecologic patients, CNR was exemplarily assessed between uterus (*ROI*_1_ ) and bladder (*ROI*_2_ ). These regions were selected, since they usually show a good CT contrast. The ROIs were in each case placed in the immediate vicinity of bladder catheter and applicator tip, respectively (Fig. 2).

## Results

**Fig. 3:**
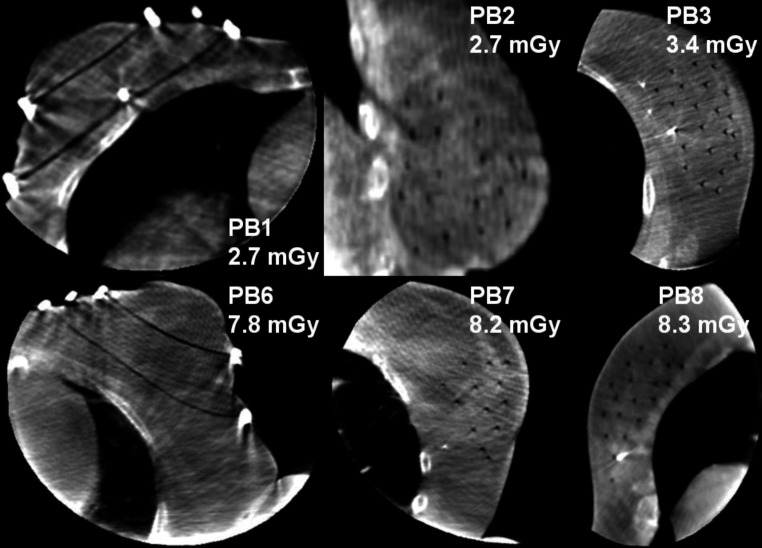
Shown are six representative control-CBCTs of breast patients. Patient IDs and CBDI_w_ as dose metric are provided. Catheters and surgical clips were distinctly identifiable in each case. Level: −40 HU, Width: 700 HU. *CBCT* cone-beam computed tomography, *Patient ID* patient identification, *CBDI*_*w*_ weighted cone-beam dose index

For six breast patients, a control-CBCT to planning-CT registration and reconstruction of catheter tracks was performed according to Kallis et al. [25]. In none of these cases, treatment adaption became necessary. All patients underwent tumor resection prior to brachytherapy and therefore had surgical clips marking the tumor bed. These were clearly identifiable on the CBCT scans (Fig. 3). A reasonable median CNR of 7.7 (range 5.9–9.2; Table 2) between catheters and surrounding tissue was obtained. Hence, the image quality was sufficient for treatment QA in each case. This was achieved at reasonable dose levels, with a median X‑ray exposure of 5.1 mGy (weighted cone-beam dose index CBDI_w_, range 2.7–8.3 mGy). One breast patient did not comply with the given breathing commands, which led to severe motion artifacts. Another patient could not be examined due to technical problems.

**Fig. 4:**
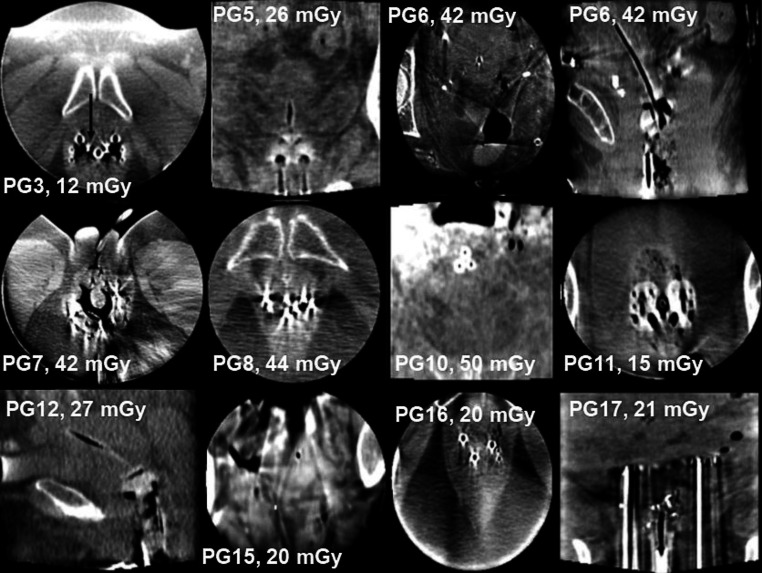
Representative control-CBCTs of gynecologic patients. The applicators were identifiable in each case. For PG3, the applicator–transfer tube assignment was checked by following the inserted X‑ray markers (*black arrow*). However, particularly in sagittal/coronal planes, no clear soft tissue differentiation was feasible in general. Significant artifacts at air–tissue transitions (e.g., PG6, PG10, PG15), directional noise (e.g., PG8, PG11, PG16), and hardening artifacts (e.g., PG3, PG8, PG17) appeared. Images were selected so that each applicator type was represented at least twice, or once if it was used once only. Level: 40 HU, Width: 500 HU

The CBCT scans of gynecologic patients allowed in general distinct visualizations of implanted applicators, due to their high contrast to the tissue (Fig. 4). The courses of vaginal and intrauterine applicators were well identifiable for the majority of patients. In each of these cases, applicator position control could be performed. In none of these cases, relevant positional changes, shifts, or slippage were identified. The deviations of the applicator and needle distances measured on the control-CBCT to the ones documented on the planning-CT are provided in Table 3 for each patient. The applicator–transfer tube assignment was feasible by following the courses of the inserted X‑ray markers (Fig. 4, PG3). However, the exact position verification of implanted needles was not always possible. This was specifically the case when needles and particularly needle tips were in close proximity to sigma or rectum filled with intestinal gas. Here, streaking and shadow artifacts originating from these air–tissue transitions substantially impaired the image quality (Fig. 4). Under these circumstances, the CBCT scans were unsuitable for a complete treatment QA. This was the case for 5 of the 18 examined patients (Table 3).

**Table 3:** Mean and maximum distance deviations between applicator/needle positions, measured between planning-CT and control-CBCT for gynecologic patients. The distances between the individual applicator/needle tips and the in-plane distances at the symphysis level were considered. We also indicated if the treatment quality assurance (QA) was significantly affected by image artifacts

ID	Mean deviation at symphysis level [mm]	Maximum deviation at symphysis level [mm]	Mean deviation between tips [mm]	Maximum deviation between tips [mm]	Needles/applicators significantly affected
PG1	0.7	1.2	0.9	1.9	–
PG2	0.9	1.6	2.0	2.7	–
PG3	0.5	1.5	0.5	0.8	–
PG4	0.4	0.8	1.2	2.3	2
PG5	0.2	0.6	2.0	2.9	–
PG6	0.4	0.7	1.3	2.8	1
PG7	0.4	0.7	0.3	0.6	2
PG8	0.7	1.5	1.1	2.9	–
PG9	0.7	1.7	0.8	1.7	5
PG10	Not measured, Heymann-Packing	Not measured, Heymann-Packing	1.4	2.4	–
PG11	0.3	0.7	1.5	2.5	–
PG12	0.9	2.0	2.0	2.7	–
PG13	0.6	1.1	1.2	2.4	–
PG14	0.5	0.8	0.7	1.3	1
PG15	0.7	1.2	0.9	1.9	–
PG16	0.5	1.0	0.8	2.8	–
PG17	0.6	1.5	1.4	2.9	–
PG18	1.1	1.3	1.5	2.3	–

*ID* identification, *PGx* Gynecologic patient number x

Although the IRm was principally suitable for validating applicator positions, the device showed distinct weaknesses in the imaging of gynecologic patients. As mentioned, severe artifacts occurred at air–tissue transitions. In addition, directional noise originating from pelvic bones and hardening artifacts in the applicators’ vicinity further impaired image quality (Fig. 4). A clear delineation of organic structures (especially of cervix, uterus, and intestine) as well as the identification of vessels and ureters was not possible in general, due to insufficient contrast differences (Fig. 4). This assessment was quantitatively confirmed by the low CNR (median 2.1, range 0.2–4.8; Table 2) measured exemplarily between uterus and bladder. Thus, despite a high median CBDI_w_ of the examinations of 26 mGy (range 9–50 mGy), individual soft tissues could not be differentiated sufficiently. Note, that the examined patients had a median BMI of 23 (range 20–36; Table 2) and thus represented in general normal weighted patients [26].

Regarding the workflow, the gantry’s large diameter allowed working free and flexible at the patient, e.g., for connecting afterloader transfer tubes. The vast FPD combined with the VDW facilitated the acquisition of FOVs sufficiently dimensioned for applicator arrangement control, as well as exact FOV adjustments even for non-isocentric patient positions. Due to its motorized mobility, the system can be maneuvered time-efficiently into imaging position regardless of the exact patient table position within the room. The total time from patient positioning (including repositioning of immobile patients from bedside) to the examination end was <5 minutes for all patients. In each case, the CBCT scans were available fully reconstructed in <50 seconds after acquisition completion. Hence, the fast assessment of all images could always be performed directly on the HMI.

## Discussion

By means of the IRm, we created a time-efficient workflow for the QA of breast and gynecologic brachytherapy. The versatile battery-powered mobility allowed fast maneuvers of the device. The reconstructed images were quickly available, which enabled direct and fast assessments regarding decisions on potential therapy adaptions. In this respect, a mobile CBCT system on-ward strongly facilitates the brachytherapy workflow, since neither patients nor staff have to be sent to more distant CT scanners. This leads particularly to the technical feasibility of performing a treatment QA also for gynecologic patients, which was previously not practicable due to the high effort involved (e.g. transport to distant CT scanners). Such a high effort in particular also precludes the acquisition of magnetic resonance imaging (MRI) scans at off-site locations. On-site MRI for interventional purposes, which would be most desirable due to the superior soft tissue delineability [27–29], is logistically still not implementable at many brachytherapy sites at all.

For none of the examined patients, the necessity for therapy adaption was diagnosed. Despite this positive experience, performing a treatment QA is considered important for therapy success. For breast patients, Kallis et al. [25] showed that treatment adaptions are indicated in 4% of all cases. For gynecologic brachytherapy, other authors already found substantial variations of the dose delivered to target volumes and OARs throughout the treatment course [10–14]. These were related to both inter- and intrafractional anatomical variations (e.g., organ and patient motion), which may lead to altered applicator positions with significant dosimetric impact [10–14]. It is one very important precondition of adaptive brachytherapy to detect such applicator arrangement changes and to adapt the treatment instantaneously to the altered conditions. In this respect, the implementation of automated procedures for treatment plan verification and immediate re-planning (in case of plan invalidity) is most desirable. Hence, this is the subject of ongoing research [10, 30, 31]. In summary, it is important for the precise therapy of patients to perform a treatment QA. CBCT represents a functional and smooth modality for this purpose.

For breast patients, the IRm’s image quality fulfilled clinical requirements at reasonable dose levels. The obtained CNR between catheters and tissue of at median 7.7 was approximately in the range of planning-CTs (median CNR of about 9, measured as described in the “Assessment of structure differentiability” section). Thus, the CBCT scans enabled distinct catheter reconstructions. Treatment QA for breast patients was therefore feasible by using the IRm scans without major drawbacks compared to conventional CT images. However, the current scan times of the IRm of 16–18 seconds are a disadvantage compared to conventional CT (acquisition time in our clinic: <6 seconds). This was also evident from the fact that one examined patient was not able to hold her breath. In particular, based on our experience, not all patients are able to follow correspondingly long breathing commands immediately after surgery. However, this would be required for the acquisition of planning-CTs. To avoid potential motion or breathing artifacts, it is therefore desired to reduce the IRm’s acquisition time. Currently, this associated with enhanced artifacts and decreased circularity of catheters due to partial undersampling. The scan time reduction with undiminished image quality is therefore subject of ongoing work.

The CBCT scans of gynecologic patients were acquired with a median CBDI_w_ of 26 mGy. This exceeds the dose of comparable devices (CBDI_w_ about 20 mGy) [32] and also conventional CT systems (CTDI_vol_ ≤15 mGy) [33, 34]. Despite this increased radiation exposure, distinct differentiations of pelvic organs and tissues were not achievable with the IRm. Target structures and OARs could only be inadequately separated. This was quantitatively confirmed by the CNRs calculated exemplarily between uterus and bladder. With a median CNR of only 2.1, the scans did generally not fulfill Rose’s criterion (Rose’s criterion states that a structure must show a CNR of at least 3 to 5 to be detectable) [35]. This was clear evidence for insufficient low-contrast differences. Please note again that the examined patients represented with a median BMI of 23 in general normal weighted patients, as reported in the “Results” section [26]. The inadequate CNR is therefore not directly attributable to extreme patient characteristics (such as it might be the case for very obese patients) accompanied by strong object scatter. Rather, it indicates a lack of CT-number contrast in the device’s imaging performance. For comparison, planning-CTs yield a respective median CNR of about 6 (again measured as described above). Furthermore, as mentioned, the IRm showed severe artifacts in the imaging of gynecologic patients.

Based on these observations, we conclude that the routine use of the IRm for the purpose of differentiating pelvic soft tissues is currently not justifiable. In particular, the IRm can currently also not support the intraoperative, image-guided insertion of applicators. This is because (due to the lack of organ differentiability) no reliable prediction can be made in which direction and how far applicators and needles can be inserted without risking injuries of corresponding OARs. In this respect, the imaging performance of the IRm is inferior to conventional CT. However, as reported, the device can be used for the verification of the implant stability in-situ and showed to be in principle applicable for the treatment QA of gynecological patients. Although for some patients (Table 3) image artifacts substantially impaired the precise detection of individual needle tips, the IRm enables imaging for treatment verification directly on the ward. This is a procedure that was previously very impractical in our hospital due to the high effort involved, as described above. We think that the advantage of such an on-site treatment verification outweighs the disadvantage of a suboptimal image quality and can have a large benefit for individual patients.

From our perspective, intraoperative CBCT-guided applicator insertion might represent an important step for adaptive gynecologic brachytherapy at many brachytherapy wards. However, to support this by the IRm, significant improvements of its imaging performance are imperative. This relates in particular to improvements in image contrast and soft tissue delineation. Nonetheless, with CBCT it appears feasible to detect the position of applicators precisely, especially around the fundus uteri or deep in the pelvis (in regions for which transrectal sonography is not or only limited applicable). For this, CBCT imaging is appropriate and beneficial. The principle of mobile, non-isocentric imaging also enables patient examinations directly in the surgical theater. This offers a possibility to improve the accuracy of implantations and consequently of treatment quality. A large gantry, as provided by the IRm, further enables imaging in the lithotomy position. This procedure was previously only possible to a limited extent with other gantry-based CBCT-CT/systems. Hence, we think that mobile CBCT imaging with the IRm has in principle high potential for image-guided adaptive brachytherapy of gynecologic malignancies. However, as mentioned, improvements of the device’s imaging performance are imperative.

## Conclusion

By means of a mobile device for on-site CBCT imaging, we implemented a time-efficient workflow for the QA of breast and gynecologic brachytherapy. However, significant performance enhancements of the IRm are strictly required for treatment planning imaging and, most desirable, intraoperative image-guidance.
